# Design and Control of a Wheeled Bipedal Robot Based on Hybrid Linear Quadratic Regulator and Proportional-Derivative Control

**DOI:** 10.3390/s25175398

**Published:** 2025-09-01

**Authors:** Yu Xu, Zhaoqiang Wang, Chenhui Lu

**Affiliations:** School of Mechanical and Automotive Engineering, Shanghai University of Engineering Science, Shanghai 201620, China; xuyu0712x@gmail.com (Y.X.); lchhuiz@163.com (C.L.)

**Keywords:** wheeled bipedal robots, four-link structure, kinematic analysis, linear quadratic regulator (LQR), motion control

## Abstract

Wheeled bipedal robots (WBRS) combine the terrain adaptability potential of legged robots with the motion efficiency of wheeled robots, but the terrain adaptability is affected by the control system. Aiming at the defect that the traditional modeling ignores the dynamic changes in head angle and center of mass height, this paper proposes a method of integrated dynamic modeling and hierarchical control. The posture balance optimizes the system performance index through the linear quadratic regulator (LQR) to control the in-wheel motor, and the state feedback matrix is designed to suppress the tipping caused by external interference. At the same time, the changes in head angle and center of mass height are included in the balance control variables. The center of mass height changes are fed back through the proportional differential (PD) control and virtual model control (VMC) algorithm to control the joint motor. Simulation experiments are carried out on multiple platforms to verify that the proposed method effectively improves the control robustness of the traditional wheeled bipedal robot through geometric-dynamic coupling modeling and LQR-PD hybrid control, providing a new method of complex terrain adaptive control.

## 1. Introduction

In recent years, robotics technology has gradually penetrated various fields, demonstrating unique potential in complex terrain exploration and surveying. Wheeled and legged robots have their respective advantages: four-wheeled robots are energy-efficient and have large payloads but are unsuitable for narrow spaces, while wheeled bipedal robots (WBRs) integrate the motion efficiency of wheeled robots and the terrain adaptability of legged robots, enabling fast movement on flat ground and stability in rough terrain [1]. With high speed, low noise, and strong maneuverability, WBRs can handle complex tasks like climbing and transporting, making them an important research direction in robotics. However, traditional modeling often ignores the dynamic changes in head angle and centroid height, resulting in insufficient adaptability of the control system in complex terrains.

Extensive research on wheel-legged robots has been conducted domestically and internationally, as follows: the Boston Dynamics’ Handle robot (Boston Dynamics, Waltham, MA, USA) uses a bionic ostrich mechanical structure and hydraulic drive, with a balance-like structure facilitating attitude control [2]; the Ascento robot from ETH Zurich (ETH Zurich, Zurich, Switzerland) realizes whole-body control through rigid-body dynamics and kinematics loops combined with the linear quadratic regulator (LQR) [3]; and the SK8O robot developed by the Czech Technical University in Prague (Czech Technical University in Prague, Prague, Czech Republic) uses LQR to control wheel torque for balance and velocity tracking, combined with the unscented Kalman filter (UKF) for angle state estimation [4]. Chen Yang et al. constructed a dynamic model and designed controllers based on LQR and VMC for wheel-legged balancing robots [5]. Additionally, the SR600 robot from Harbin Institute of Technology (Harbin Institute of Technology, Harbin, China) adopts PID control and passes ROS-Gazebo simulation verification [6,7], while Tencent Robotics X Lab’s (Tencent Robotics X Lab, Shenzhen, China) Ollie robot uses a nonlinear controller based on Interconnection and Damping Distribution-Passive Control (IDA-PBC) to achieve energy-minimized state velocity tracking [8,9]. Traiko Dinev proposed a variable-length wheeled inverted pendulum template model, combined with model predictive control, to achieve dynamic motion planning (such as jumping and swinging) for wheeled jumping robots [10]. Lu et al. [11] focused on trajectory planning for jumping and soft landing of a new wheeled bipedal robot, providing insights to enhance the robot’s ability to execute complex movements. Zhao et al. [12] dedicated efforts to improving the dynamic performance of wheeled bipedal robots through bio-inspired mechanical design and the development of novel controllers. Raza et al. [13] enhanced the balance stability of wheel-legged biped robots by means of arm acceleration control. In addition, Aydogmus and Yilmaz [14] conducted a comparative analysis of reinforcement learning algorithms for bipedal robot locomotion, providing a reference for the selection of control algorithms; Chen et al. [15] studied underactuated motion planning and control for jumping of wheeled bipedal robots; Yu et al. [16] carried out modeling for pose tracking of wheeled bipedal robots based on model predictive control (MPC); Tang et al. [17] proposed a comprehensive planning and control strategy for the somersaulting jump of wheeled bipedal robots; Liu et al. [18] explored the terrain adaptability and in situ transformation of wheeled bipedal robots; Dong et al. [19] studied the equivalent predictive control and handle point control for bipedal vehicle transformable robots under various disturbances; Lu et al. [20] proposed a planning and control scheme for the run-and-jump motion of a wheeled bipedal robot considering dynamic constraints; and Chen et al. [21] designed and implemented a novel two-wheeled composite self-balancing robot for stationary operations in unknown terrain.

Notably, significant progress has been made in advanced control technologies for wheeled bipedal robots in recent years. Mohammed Yousri Silaa et al. proposed innovative control strategies, as follows: on one hand, they employ stochastic gradient descent to adaptively tune sliding mode control gains online, significantly optimizing trajectory tracking performance for robot manipulators [22]; on the other hand, they combine neural-network-based system identification with discrete extended Kalman filtering for state estimation, effectively improving the accuracy of trajectory tracking control for wheeled mobile robots [23]. However, existing studies exhibit notable deficiencies in dynamic coupling modeling of centroid height and head angle, making it difficult to achieve effective balance between motion stability and complex terrain adaptability.

Robot control in dynamic and uncertain environments relies not only on local feedback mechanisms, but also on system-level risk robustness design. As Sajib et al. (2025) [24] pointed out through comprehensive risk assessment modeling of passenger ferry accidents, the reliability of safety-critical systems depends on the dual optimization of structural vulnerabilities and procedural defects. In unstructured terrains, robots may face multi-dimensional risks such as sudden terrain changes, sensor noise, and actuator failures. Their control frameworks must simultaneously satisfy real-time feedback adjustment (e.g., the LQR-PD hybrid control in this paper) and system-level risk buffering (e.g., dynamic fault diagnosis and fault-tolerant strategies) to achieve robust and stable operation.

This paper employs the four-link series structure in the literature [4], which has fewer drive motors, lower control difficulty, and larger load space. By designing a 2D linkage mechanism with Pyslvs to determine link lengths, a geometric-dynamic coupling modeling and an LQR-PD hybrid control method are proposed, as follows:

LQR is used to optimize system performance indices, and a state feedback matrix is designed to suppress any tipping caused by external disturbances; head angle and centroid height changes are incorporated into balance control variables, with centroid height feedback adjusted by PD control and virtual model control; and simulations on multiple platforms (e.g., Webots) verify that the method effectively improves the control robustness of traditional WBRs, providing a new approach for complex terrain adaptive control.

## 2. System Modeling

The wheeled bipedal robot is a complex nonlinear multiple-input multiple-output system, which regards the robot as a combination of left and right legs, left and right wheels, plus the body, and left and right symmetry, so an idealized setting can be made.

**Figure 1:**
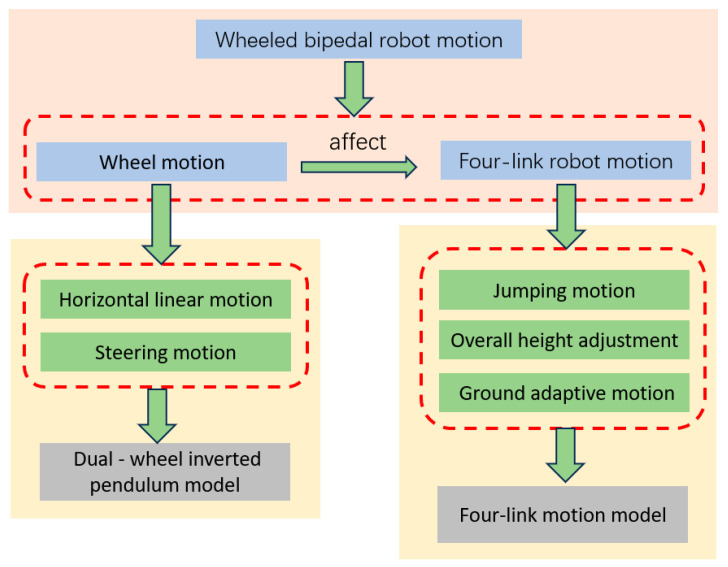
Block diagram of robot dynamics modeling and analysis.

The robotic dynamic model is decomposed into wheel motion and leg motion. The wheel subsystem, responsible for linear locomotion and steering, is simplified as a two-wheel inverted pendulum model. The leg subsystem employs a four-bar linkage mechanism to achieve jumping, full-body height adjustment, and adaptive locomotion on uneven terrain. By focusing on analyzing the dynamic model of the four-bar linkage motion, the originally coupled complex model is streamlined. As shown in Figure 1, this decoupling strategy simplifies the design process while retaining critical functionalities, thus enabling efficient control of the robot’s hybrid mobility modes.

### 2.1. Dual-Wheel Inverted Pendulum Model

**Figure 2:**
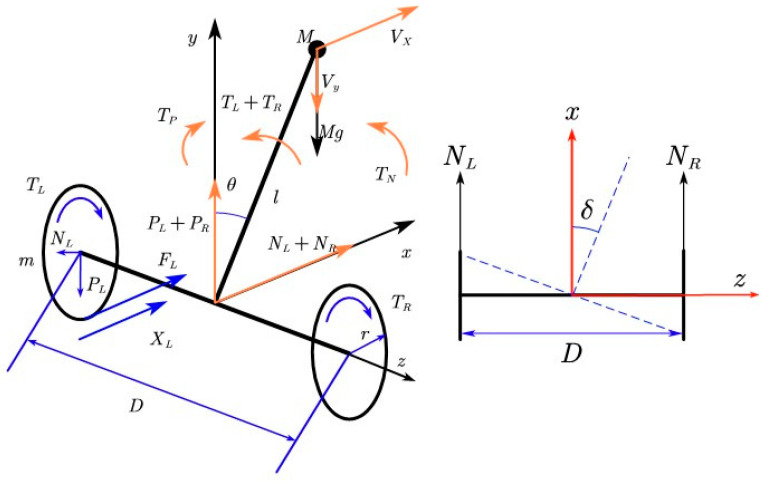
Two-wheeled inverted pendulum model.

The mass of the body is equivalently concentrated in the position of the center of mass. Ignoring the effect of the leg motion on the wheeled movement and the mass of the four links, the driving wheels are in non-slip rolling friction with the ground. Therefore, the four-link wheel-legged robot can be equivalently simplified into a two-wheeled inverted pendulum model with variable rod length [25], see Figure 2 and Table 1.

The force balance equation of the left wheel along the *x*-axis is:

(1) $$m{\ddot{x}}_{L}=F_{L}-N_{L}$$

The left wheel torque balance equation around the axle is:

(2) $$I{\dot{\omega }}_{L}=T_{L}-F_{L}r$$

(3) $$I{\dot{\omega }}_{L}=T_{L}-r(m{\ddot{x}}_{L}+N_{L})$$

The formula for the angular acceleration of wheel rotation versus the acceleration of the wheel axle is:

(4) $${\dot{\omega }}_{L}=\frac{{\ddot{x}}_{L}}{r}$$

Substitute Equation (4) into Equation (3) to calculate the dynamics equation for the left wheel, as follows:

(5) $$\left(m+\frac{I}{r^{2}}\right){\ddot{x}}_{L}=\frac{T_{L}}{r}-N_{L}$$

Similarly, the dynamics equation of the right wheel can be calculated as follows:

(6) $$\left(m+\frac{I}{r^{2}}\right){\ddot{x}}_{R}=\frac{T_{R}}{r}-N_{R}$$

The acceleration of the whole body can be calculated from the acceleration of the left and right wheels as follows:

(7) $$\ddot{x}=\frac{{\ddot{x}}_{L}+{\ddot{x}}_{R}}{2}$$

Sorting Formulas (5)–(7) can be carried out as follows:

(8) $$\left(m+\frac{I}{r^{2}}\right)\ddot{x}=\frac{T_{L}+T_{R}}{2r}-\frac{N_{L}+N_{R}}{2}$$

The body part can be regarded as an inverted pendulum in the plane composed of the *x*-axis and *y*-axis, the force analysis of the body center of mass, the force sum of the whole machine $N_{L}+N_{R}$ and $P_{L}+P_{R}$ can be moved to the center of mass, and the additional couple sum $T_{N}\;\;and\;\;T_{P}$ can be calculated as follows:

(9) $$T_{N}=\left(N_{L}+N_{R}\right)lcos\theta ,T_{P}=\left(P_{L}+P_{R}\right)lsin\theta$$

Change the velocity at the center of mass to V and V as follows:

(10) $$v_{x}=\dot{x}+l\dot{\theta }cos\theta ,v_{y}=l\dot{\theta }sin\theta$$

The force balance equation for the center of mass on the *x*-axis is:

(11) $$M{\dot{v}}_{x}=N_{L}+N_{R}$$

(12) $$M(\ddot{x}+l\ddot{\theta }cos\theta -l{\dot{\theta }}^{2}sin\theta )=N_{L}+N_{R}$$

The force balance equation for the center of mass on the *y*-axis is:

(13) $$M{\dot{v}}_{y}=Mg-(P_{L}+P_{R})$$

Combine (10), (13) to obtain the following:

(14) $$Mg-M\left(l{\dot{\theta }}^{2}cos\theta +l\ddot{\theta }sin\theta \right)=P_{L}+P_{R}$$

The moment balance equation of center of mass around *z*-axis is:

(15) $$J_{z}\ddot{\theta }=T_{P}-T_{N}-(T_{L}+T_{R})$$

By combining Equations (9), (12), (14) and (15), the dynamic equations for the body part of the whole machine are calculated as follows:

(16) $$\left(J_{Z}+Ml^{2}\right)\ddot{\theta }=Mglsin\theta -M\ddot{x}lcos\theta -(T_{L}+T_{R})$$

By substituting Equation (12) into Equation (8), the dynamic equations of the wheel section of the whole machine can be calculated as follows:

(17) $$\left(M+2m+\frac{2I}{r^{2}}\right)\ddot{x}=\frac{\left(T_{L}+T_{R}\right)}{r}-Ml\ddot{\theta }cos\theta +Ml{\dot{\theta }}^{2}sin\theta$$

When the pitch inclination of the body is small, the robot maintains its balance only for a smaller angle, performing linearization calculations as follows:

(18) $$cos\theta =1,sin\theta =\theta ,{\dot{\theta }}^{2}=0$$

Combining Equations (16)–(18), the linearized set of equations for the dynamics of plane motion can be calculated as follows:

(19) $$\left\{\begin{array}{c} r\left(M+2m+\frac{2l}{r^{2}}\right)\ddot{x}=(T_{L}+T_{R})-Mrl\ddot{\theta }\\ (J_{z}+Ml^{2})\ddot{\theta }=Mgl\theta -M\ddot{x}l-(T_{L}+T_{R})\; \end{array}\right.$$

The steering of the whole machine adopts the method of two-wheel speed differential, and the moment balance equation of the whole machine around the *y*-axis is:

(20) $$J_{y}\ddot{\delta }=(N_{L}-N_{R})\frac{D}{2}$$

The relationship between the course angular acceleration of the whole machine and the wheel acceleration is:

(21) $$\ddot{\delta }=\frac{{\ddot{x}}_{L}-{\ddot{x}}_{R}}{D}$$

After sorting out Equations (8), (20) and (21), the dynamic equation of steering motion can be obtained by calculating the following:

(22) $$\ddot{\delta }=\frac{T_{L}-T_{R}}{r(mD+\frac{ID}{r^{2}}+\frac{2J_{y}}{D})}$$

### 2.2. Four-Link Motion Model

The whole machine is symmetrical on the left and right, the kinematics of the four-link structure of the left leg are analyzed in detail, and the relevant parameters are illustrated in Table 2.

The four-link geometrical relationship was analyzed to obtain the pendulum height (the vertical angle between the drive wheel axis and the joint) by using the initial state of the joint motor, inputting $\theta_{1}$, and calculating the auxiliary linkage (the distance from the joint at the high point to the intersection point of the lower bar), as well as the angle between the individual links [26].

(23) $$l_{5}=\sqrt{l_{2}^{2}+l_{4}^{2}-2\cdot l_{2}\cdot l_{4}\cdot \cos\left(\theta_{1}+\frac{\pi }{4}\right)}$$

(24) $$\theta_{2}=\arcsin\left(\frac{l_{4}\cdot \sin\left(\theta_{1}+\frac{\pi }{4}\right)}{l_{5}}\right)$$

(25) $$\theta_{3}=\arccos\left(\frac{l_{23}^{2}+l_{5}^{2}-l_{3}^{2}}{2\cdot l_{23}\cdot l_{5}}\right)$$

(26) $$\theta_{4}={\pi -\theta }_{1}-\theta_{2}-\theta_{3}$$

(27) $$l_{leg}=l_{2}\cdot \sin\left(\theta_{1}\right)+l_{1}\cdot \sin\left(\theta_{4}\right)$$

Then, inputting the height of the pendulum rod can be reversed to find $\theta_{1}$, which can get $\theta_{5}$ and $\theta_{6}$, as follows:

(28) $$\theta_{1}=arcsin\left(\frac{l_{leg}^{2}+l_{2}^{2}-l_{1}^{2}}{2\cdot l_{leg}\cdot l_{2}}\right)$$

(29) $$\theta_{5}=\pi -\theta_{4}$$

(30) $$\theta_{6}=\arcsin\left(\frac{l_{5}\cdot \sin\left(\theta_{3}\right)}{l_{3}}\right)-\theta_{4}$$

Find the vertical coordinates of each link after knowing each angle, and then find the total center of gravity height $y_{c}$, as follows:

(31) $$x_{c2}=\frac{l_{2}\cdot \sin\left(\theta_{1}\right)}{2}$$

(32) $$x_{c1}=l_{2}\cdot sin\left(\theta_{1}\right)+\frac{l_{1}\cdot sin\left(\theta_{5}\right)}{2}$$

(33) $$x_{c3}=-l_{4}\cdot \sin\left(\frac{\pi }{4}\right)+\frac{l_{3}\cdot \sin\left(\theta_{6}\right)}{2}$$

(34) $$x_{c23}=l_{2}\cdot \sin\left(\theta_{1}\right)-\frac{l_{23}\cdot \sin\left(\theta_{4}\right)}{2}$$

(35) $$L_{0}=\frac{m_{1}\cdot x_{c1}+m_{2}\cdot x_{c2}+m_{3}\cdot x_{c3}+m_{23}\cdot x_{c23}}{m_{1}+m_{2}+m_{3}+m_{23}}$$

The final center of gravity height of the output pendulum, $l_{w}$, is the overall center of mass offset of the system:

(36) $$l_{w}=l_{leg}-L_{0}$$

The distance of the total center of gravity of the system relative to the axis of the drive wheels, expressed as a weighted average of the center of mass of the body and the center of mass of the pendulum, is:

(37) $$L_{1}=\frac{\left(l_{b}\cdot m_{b}+l_{w}\cdot m_{l}\right)}{m_{p}}$$

The moment of inertia of the drive wheel there is:

(38) $$I_{w}=m_{w}\cdot R_{1}^{2}$$

The total rotational inertia of the system (body and pendulum) there is:

(39) $$I_{p}=m_{b}\cdot (l_{b}-L_{1}{)}^{2}+m_{l}\cdot (L_{1}-l_{w}{)}^{2}$$

The effect of the pendulum on the normal force of the system contains the horizontal and rotational acceleration components at the end of the pendulum, i.e., the net force in the x direction:

(40) $$N=m_{p}\cdot \frac{d^{2}x+L\cdot sin(\theta )}{dt^{2}}$$

The gravitational force of the system and the acceleration component along the vertical axis, i.e., the net force in the Y direction, are mainly used to describe the dynamic effects of the pendulum in the vertical direction, as follows:

(41) $$P=m_{p}\cdot g+m_{p}\cdot \frac{d^{2}(L\cdot cos(\theta ))}{dt^{2}}$$

Describing the dynamics of the horizontal motion of the driving wheel, the linear acceleration equation is obtained from the joint action of the driving moment T and the normal force N as follows:

(42) $$\frac{d^{2}x}{dt^{2}}=\frac{T-N\cdot R}{\frac{I_{w}}{R}+m_{w}\cdot R}$$

The angular motion dynamics of the system are affected by the combined gravitational force P normal force N, and driving moment T, as follows:

(43) $$I_{p}\cdot \frac{d^{2}\theta }{dt^{2}}=P\cdot L\cdot \sin\left(\theta \right)-N\cdot L\cdot cos(\theta )-T$$

## 3. Analysis of Control Algorithm

Aiming at the nonlinear and strongly coupled characteristics of wheeled bipedal robots, this section analyzes the design and collaborative mechanisms of LQR, PD control, VMC, and linear fitting algorithms to achieve posture balance, centroid adjustment, and anti-interference control.

### 3.1. Linear Quadratic Regulator (LQR) Control

The equation of state is linearized by expanding $\theta ,\dot{\theta },x,\dot{x}$ (angle, angular velocity, displacement, and velocity) and the input moment T around the equilibrium point $\left(\theta =0,\dot{\theta }=0,x=0,\dot{x}=0\right)$ and neglecting higher order terms [27].

The linearized equation has the derivatives of $\theta ,\dot{\theta },x,\dot{x}$ for the Jacobi matrix A, as follows:

(44) $$A=\left|\begin{array}{cccc} \dot{\theta } & f_{1} & \dot{x} & f_{2}\\ \theta_{0} & \dot{\theta } & x_{0} & \dot{x}\\ \theta_{0} & \dot{\theta } & \dot{x} & T\\ 0 & 0 & 0 & 0 \end{array}\right|$$

Find the Jacobi matrix B for the derivatives of the input T, as follows:

(45) $$B=\left|\begin{array}{cccc} \dot{\theta } & f_{1} & \dot{x} & f_{2}\\ T & 0 & 0 & 0\\ \theta_{0} & \dot{\theta } & \dot{x} & T\\ 0 & 0 & 0 & 0 \end{array}\right|$$

The linear state space equation of the system is obtained:

(46) $$\dot{x}=Ax+Bu$$

LQR designs the feedback gain matrix K to minimize the cost function J by minimizing the following quadratic objective cost function, where Q is the state weight matrix used to adjust the importance of the state variables and R is the control weight matrix used to adjust the cost of the control inputs [28,29,30,31]:

(47) $$J=\int_{0}^{\infty }\;\left(x^{T}Qx+u^{T}Ru\right)dt$$

By using a call to the LQR (A, B, Q, R) function, the feedback gain matrix K is obtained, and K is used to control the relationship between the input u and the state variables, i.e., the system can be stabilized to the target state by controlling u through feedback.

(48) u=−K⋅x

To solve the optimal feedback gain matrix K, the LQR algorithm has to solve the algebraic Riccati equation. For discrete-time systems, it is necessary to discretize the continuous-time system, and, assuming that the discretization step is $T_{s}$, the discrete-time system can be expressed as follows:

(49) $$x_{k+1}=Fx_{k}+Gu_{k}$$

(50) $$F=e^{AT_{s}}$$

(51) $$G=\int_{0}^{T_{s}}\;e^{A\tau }Bd\tau$$

The algebraic Riccati equation is expressed as:

(52) $$F^{⊤}SG[R+G^{⊤}SG{]}^{-1}G^{⊤}SF+S-F^{⊤}SF-Q=0$$

F and G are the state space matrices of the discrete-time system; Q and R are the weight matrices; S is the solution of the Riccati equation, representing the state covariance matrix of the system; and S is a symmetric matrix. Therefore, it is assumed that:

(53) $$S=\left[\begin{array}{cccc} s_{11} & s_{12} & s_{13} & s_{14}\\ s_{12} & s_{22} & s_{23} & s_{24}\\ s_{13} & s_{23} & s_{33} & s_{34}\\ s_{14} & s_{24} & s_{34} & s_{44} \end{array}\right]$$

By substituting F, G, Q, and R into the Riccati equation, nonlinear equations about S are obtained by the iterative method, and S is solved.

The optimal feedback gain matrix K can be obtained by solving the Riccati equation, as follows:

(54) $$K=[R+G^{⊤}SG{]}^{-1}G^{⊤}SF$$

### 3.2. PD Control

In this model, the actual leg length $l_{leg}$ of the whole machine is calculated according to Equation (35). The expected leg length $L_{desired}$ of the robot is set, so the height error e between the actual leg length and the expected leg length can be calculated.

(55) $$e=l_{leg}+L_{desired}$$

Then, the joint input force compensation value is calculated from the height error e by PD control (proportional-differential control).

(56) $$\tau_{PID}=K_{p}e+K_{d}\frac{de}{dt}$$

The mass of the whole machine is a component of the forces on the joints, the support of the robot on the ground is proportional to the gravitational force on the robot, and, due to the angle, the actual force is reduced. $\cos\left(\theta_{1}\right)$ reflects the effect of the angle of rotation of a joint on the force of gravity.

(57) $$\tau_{gravity}=mg\;\cos\left(\theta_{1}\right)$$

(58) $$F_{0}=\tau_{gravity}+\tau_{PID}$$

PD control is used to calculate the external force $F_{0}$ that needs to be applied on the joint motor. Omitting the I term can avoid the situation of integral saturation overshooting and improve the dynamic response speed, which is suitable for highly dynamic scenarios. The integral term will accumulate noise or high-frequency interference, which will lead to jitter of the control signal, while the differential term has a natural suppression effect on high-frequency noise, and PD control is more anti-interference.

### 3.3. Virtual Model Control

Virtual model control (VMC) is used to calculate the joint output torque, assuming that a “virtual model” exists in the system, and to map the actual mechanical properties of the system into the virtual model, i.e., the output torque of the joint obtained from the model predictive control and the output of the leg-length controller are transformed into the output torque of the joint motor [32].

**Figure 3:**
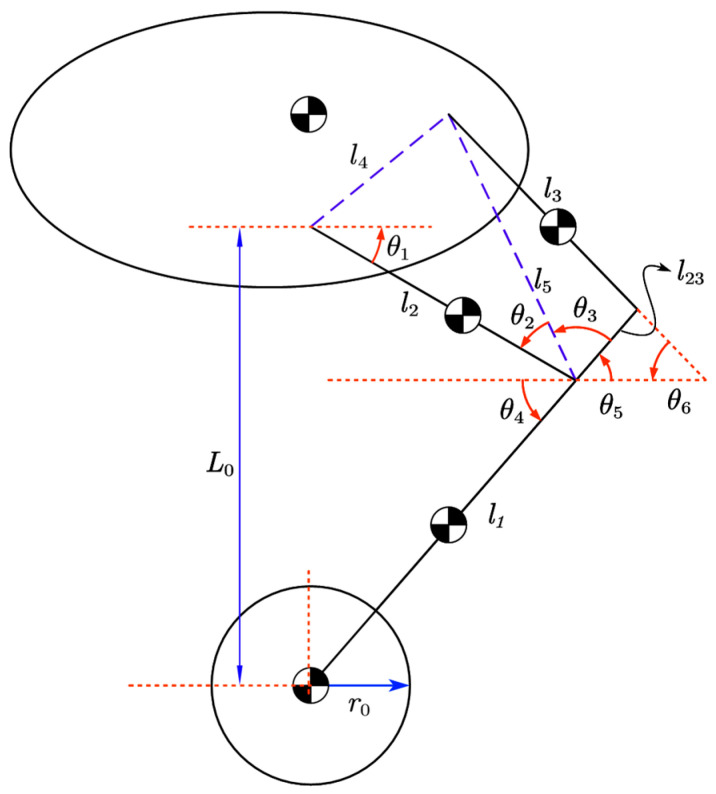
Detailed analytical model of the four-link structure.

The four-link structure of Figure 3 is analyzed by transforming the joint angles and lengths, inputting the force $F_{0}$, angle θ, and link length l, and combining them to produce a scalar J of the Jacobi matrix.

(59) $$J=l_{2}cos\left(\theta_{1}\right)+l_{1}cos\left(\theta_{1}+\alpha +\beta \right)$$

$l_{2}cos\left(\theta_{1}\right)$ denotes the position component caused by link $l_{2}$, and $\;l_{1}cos\left(\theta_{1}+\alpha +\beta \right)$ denotes the indirect position component caused by link $l_{1}$ and geometric constraints.

The angle relation α between the links can be calculated by using the arcsine theorem. Another angle relation β can be obtained by using the arcsine theorem.

The external force $F_{0}$ acting from the end-effector is the input variable in the virtual model, and the geometrical parameters and kinematic relations of the system are mapped to the joint torques through the Jacobi matrix scalar J. The system is then mapped to the joint torques.

(60) $$T_{joint}=F_{0}\cdot J$$

### 3.4. Linear Fitting

In the kinematic analysis of a four-link wheel-legged robot, the relationship between the pendulum length ($l_{leg}$) and the center of gravity height ($l_{w}$) directly affects the stability and kinematic performance of the robot. In addition, the direct calculation of the center of gravity heights under different pendulum lengths would be very complicated and computationally intensive, so the relationship between the two is modeled using a quadratic polynomial fitting method, which can simplify the subsequent computational process and quickly predict the change in the center of gravity height under different center of gravity conditions at different pendulum lengths.

Based on a predetermined range of pendulum lengths ($l_{leg}$), the height of the center of gravity corresponding to each length is calculated by traversing a series of pendulum length values. A quadratic polynomial was fitted to the computed results using the least squares method to simplify the relationship between pendulum length and center of gravity height to a fitted function.

(61) $$l_{w}=ak_{1}\cdot (l_{leg}{)}^{2}+ak_{2}\cdot l_{leg}+ak_{3}$$

The fitting parameters ak are:

(62) $$ak=[a_{1},a_{2},a_{3}]$$

(63) $$l_{w}=a_{1}\cdot (l_{leg}{)}^{2}+a_{2}\cdot l_{leg}+a_{3}$$

The relationship between the pendulum length and the center of gravity height is simplified as a quadratic polynomial, and this fitting model can visualize the trend of the system’s center of gravity height when the pendulum length increases. The center of gravity heights under different pendulum lengths can be quickly calculated without the need for complex geometric and kinematic calculations each time when performing robot control strategy design and simulation validation. For example, in LQR and PD control, accurate center of gravity heights are crucial for designing the appropriate feedback gain matrices and compensation moments, and the fitted model thus holistically improves the control system’s stability and response speed.

## 4. Modeling Simulation Verification

The effectiveness of the control algorithm is verified through multi-platform simulations, including four-link structure parameter optimization, Simulink modeling, and Webots physical simulation.

### 4.1. Definition of Connecting Rod Structure Parameters

**Figure 4:**
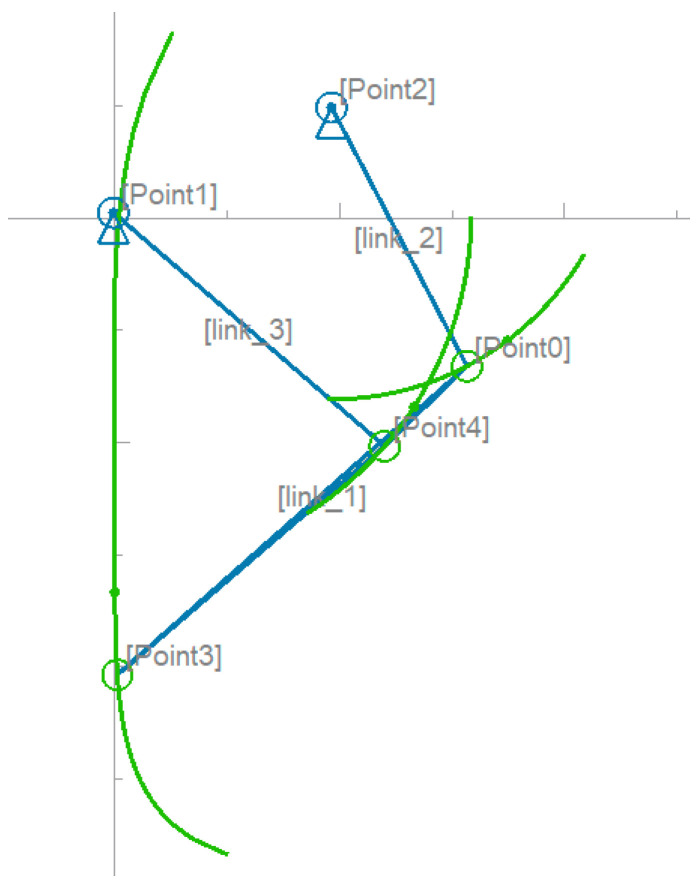
Modeling of four-link Pyslvs.

In Pyslvs-UI-22.07 (2D connecting rod structure design software), two fixed points and three movable points are established and connected together in a four-link fashion. Moreover, a virtual motor is added to one fixed point to observe the trajectory of the movable point at the end of the connecting rod, and the trajectory can be changed by setting the distance between each point, i.e., the length of the connecting rod [33]. As shown in Figure 4, at the position of the center of the wheel, at the end of the four links extending out, we set the length of the rods as 163, 164, 170, 80, and 40 and plotted the observed trajectory at this scale. The trajectory of the end in the movable space converges most closely to a straight line.

### 4.2. Simulink Simulation Modeling

To verify the feasibility of the LQR-PD controller, the model is designed and built in Simulink. The linear state space equation of Equation (46) is used to invoke the cost function J and design K, and the parameters are designed as:

(64) $$Q=\left[\begin{array}{cccc} 500 & 0 & 0 & 0\\ 0 & 1 & 0 & 0\\ 0 & 0 & 800 & 0\\ 0 & 0 & 0 & 150 \end{array}\right],R=100$$

For PD control of the whole machine height, set kp = 280, kd = 50.

### 4.3. Slope Simulation Test

The setting allows the robot to travel normally at a desired speed of 3 m/s, passing through a 20° slope and stopping.

**Figure 5:**
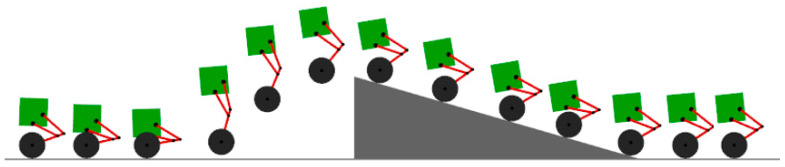
Robot over-slope state.

**Figure 6:**
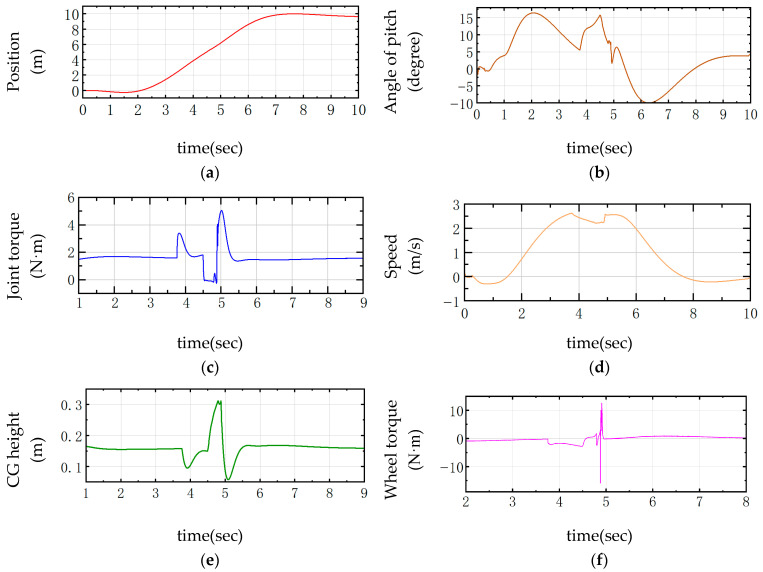
Robot control curve through the slope. (**a**) Position; (**b**) Angle of pitch; (**c**) Joint torque; (**d**) Speed; (**e**) Center of gravity height; (**f**) Wheel torque.

The over-slope state is shown in Figure 5. From the displacement and velocity change curves shown in Figure 6, it can be seen that, in the state of forward traveling, it is necessary to fall backward for a certain distance in the starting 0–2 s and then accelerate forward again. As shown in Figure 6, the change in the center of gravity first decreases with the contraction of the joints during the process of going up the slope and then increases after going up the slope. When falling to the ground, the height of the center of gravity is minimized, and the torque of the joint motors will increase in the uphill slope, mainly in the moment of contacting the ground, which requires the joint motors to drive the linkage mechanism to contract for cushioning, with a sudden increase in torque, up to 5 N·m, and the torque of the wheel motor reaches up to 13 N·m.

**Figure 7:**
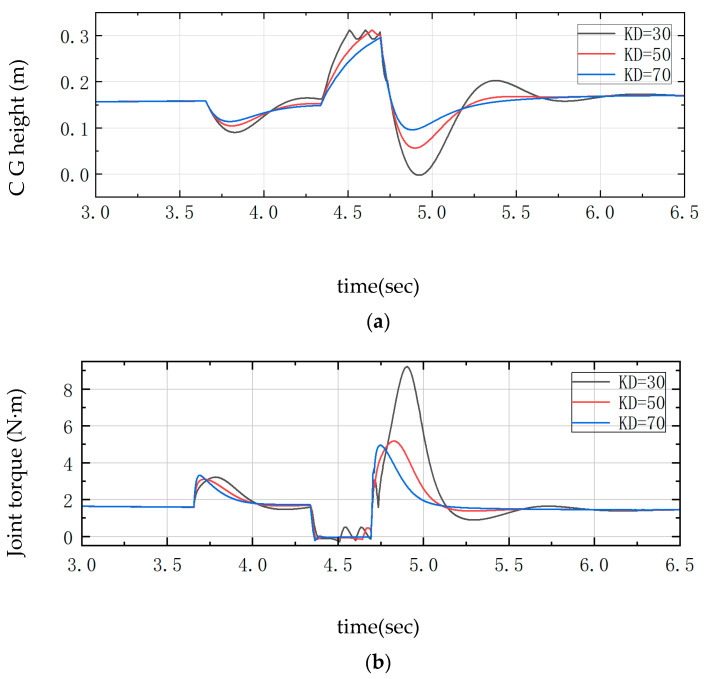
Comparison curve of over-slope differential change. (**a**) Center of gravity height; (**b**) Joint torque.

The required external input force to control the joint motors is shown in Equations (55)–(58). In the PD controller, the value of KD is adjusted to compare the joint moments with the center of gravity height. During the process of up-slope and landing buffer, as shown in Figure 7, between 3.5 s and 4.0 s, KD is too small, which will lead to a slower corresponding system, too much undulation and instability between 4.5 s and 5.0 s, and a large overshooting oscillation is visible after 5.0 s. When KD is too large, the response is too fast, and the change in the height of the center of gravity is very small, which will lead to the robot being not smooth enough to pass through the complex environment, which is a major concern in the actual test. In the actual test, there is some damage to the structural strength of the whole machine and the joint motors. Therefore, it is more appropriate to take KD = 50.

**Figure 8:**
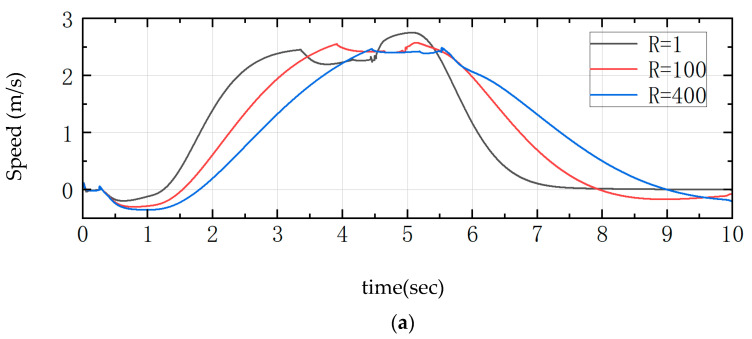

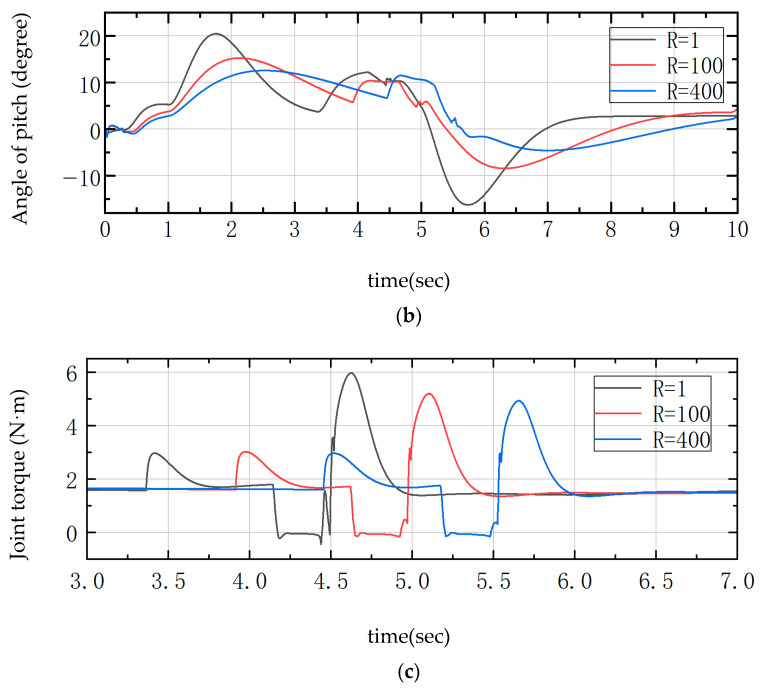
Comparison curves for changes in weights R. (**a**) Speed; (**b**) Angle of pitch; (**c**) Joint torque.

In the LQR controller, the weight R parameter (R = 1, R = 100, R = 400) is designed for the control input u. As shown in Figure 8, when R = 1, the fluctuation of the inclination angle of the whole machine is larger, and there are large peaks and valleys, with the maximum range of 17° to 21°, whereas with R = 100, the range of the inclination angle of the whole machine is from −7 to 15°, and the fluctuation is reduced by 45%. A smaller value of R makes the system less penalized by the control inputs, and the controller will tend to apply a larger torque to quickly reach the desired state. The R increases to 100 and 400, increasing the penalty for the control input and becoming more conservative in the adjustment process, the fluctuation amplitude of the joint torque gradually decreases, and the curve becomes smoother. When R is 400, although very stable, it will show the slowest response speed, a balanced response speed, and stability. R = 100 is the best, as the response time is 0.6 s shorter than that of R = 400.

### 4.4. Jump Simulation Test

**Figure 9:**
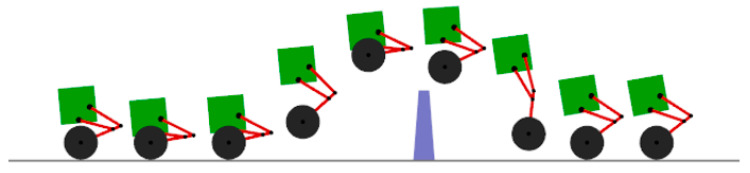
Robot jumping obstacle state.

The robot is set to run for 10 s, with a desired speed of 3 m/s, jumping at 3.3 s, and stopping in place at 7 s. The results are shown in Figure 9.

**Figure 10:**
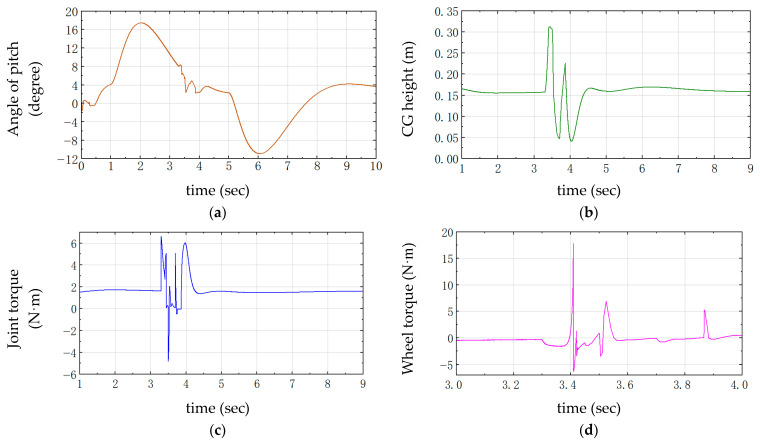
Robot jumping obstacle control curve. (**a**) Angle of pitch; (**b**) Center of gravity height; (**c**) Joint torque; (**d**) Wheel torque.

The obstacle jumping process is shown in Figure 9. The robot accelerates to 3.3 s for jumping, and, at the moment of jumping, the torque changes abruptly at 3.3 s, the joint torque reaches 6.5 N·m, and the wheel motor torque reaches 17.5 N·m, as shown in Figure 10. When the center of gravity height is jumping up, the four connecting rods are stretched to the maximum, then, when reaching the top of the jump, the connecting rods are contracted to the minimum. During the descending process, the connecting rods are gradually stretched to increase the cushioning distance. After contacting the ground, the connecting rods act as cushioning and are compressed to the minimum. Comparing the center of gravity height change curve and the joint moment curve, it is a perfect match, and the maximum height of the center of mass in the process of jumping reaches 30 cm, subtracting the distance from the center of mass at the highest point to the wheel part of 15 cm, and the whole machine can realize jumping the highest 15 cm obstacle. From the start of jumping in 3.3 s, the wheel part of the robot leaves the ground to land in 3.8 s, and the furthest distance of the jump is 1.2 m; moreover, observing the inclination curve of the whole machine in Figure 10 in the range of 3–5 s, it can be found that the robot is extremely stable. The stability of the robot is very strong. After jumping, the maximum range of the inclination angle of the whole machine is within 5°, and, after contacting the ground, it immediately maintains a balanced state, accelerates forward to an inclination angle of 5°, and then decelerates and stops.

**Figure 11:**
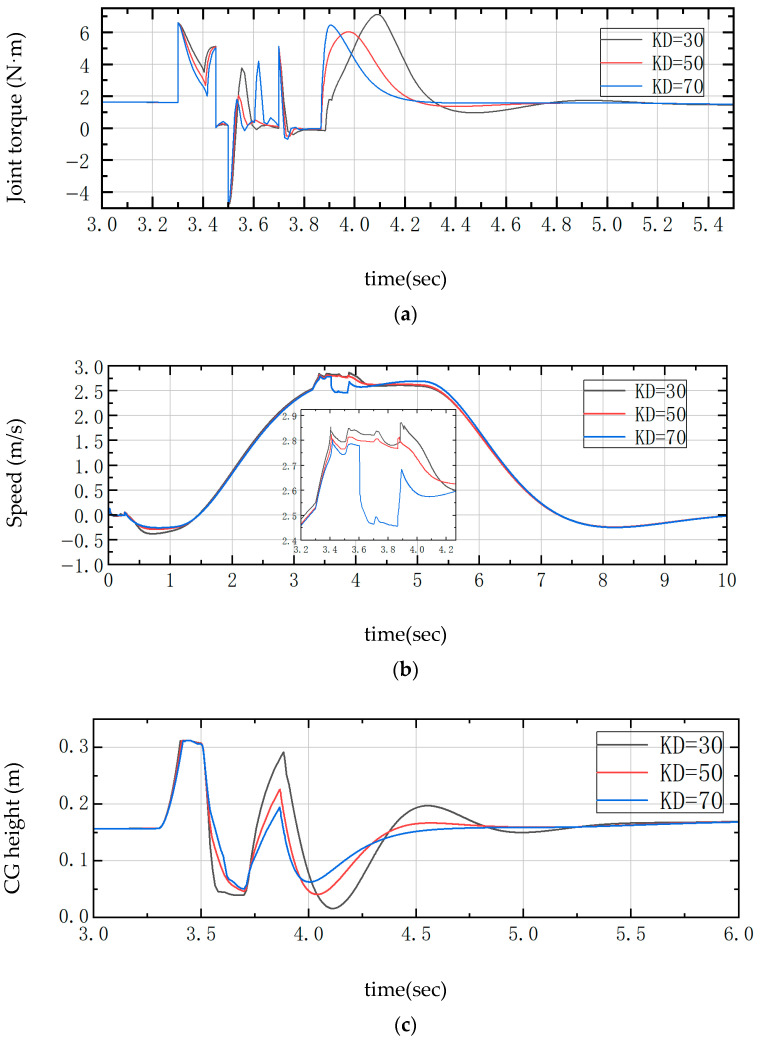

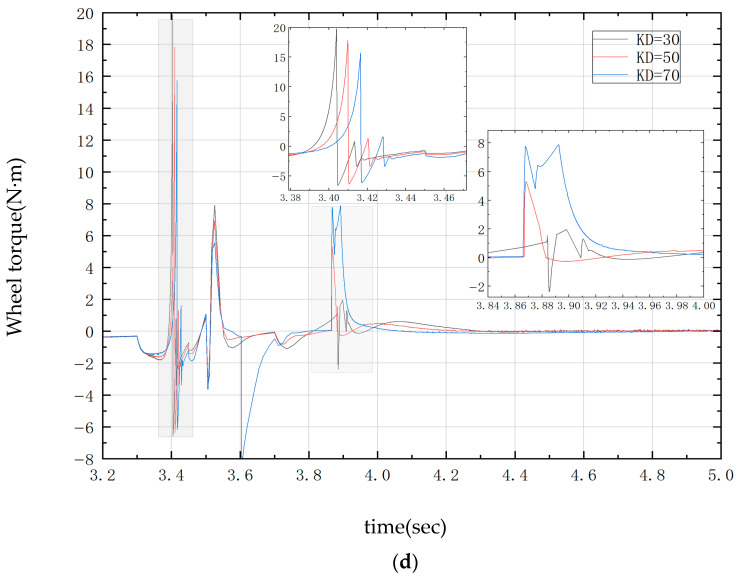
Comparison curves of differential changes in the jump state. (**a**) Joint torque; (**b**) Speed; (**c**) Center of gravity height; (**d**) Wheel torque.

During the jumping process of the designed robot, the PD is set to the jump up phase (3.3–3.6 s), as shown in Figure 11a,b,d. The larger the KD is, the larger the joint torque is, and the smaller the wheel motor torque is, which leads to a greater change in velocity. In the descending landing phase (3.8–4.2 s), observing the wheel motor torque in Figure 11c,d, the value of KD is 70, which will lead to greater oscillation, and the torque of KD is 30, which is too small to adjust the center of gravity as soon as possible, which can lead to overshooting at the same time of instability. The height of the center of gravity corresponding to KD = 30 will change up to 28 cm due to the long cushioning distance, the stability will deteriorate, and it takes 1.5 s to stabilize. While KD = 50, the change range is 18 cm and is kept stable for 0.5 s, the buffer distance is shortened by 36%, and the stabilization time is shortened by 33%; therefore, the KD is set to 50 to make the jumping process more stable.

LQR controllers are capable of handling multiple state variables and controlling inputs simultaneously, and they are suitable for complex multiple-input multiple-output (MIMO) systems. Wheel-legged robots need to simultaneously control multiple variables such as torque, body attitude, and speed of multiple joints when crossing slopes and jumping. The state space model considers these variables in a unified way to design a coherent control strategy with high dynamic adaptability. In contrast, PID controllers are suitable for single-input single-output (SISO) systems, which require the design of a system for each parameter and can also lead to mutual interference between parameters. PID controllers are less robust and sensitive to changes in model parameters. For a four-link wheel-legged robot system, small changes in the PID parameters can lead to system instability or control performance degradation, and it is only used to design external forces acting on a single-input single-output articulated motor. The LQR controller takes into account the geometrical model of the system and the performance metrics and has better robustness to changes in the model parameters. The LQR controller maintains good control performance even when there are errors in the model parameters or changes in the dynamic characteristics of the system.

## 5. Webots Simulation Experiment

**Figure 12:**
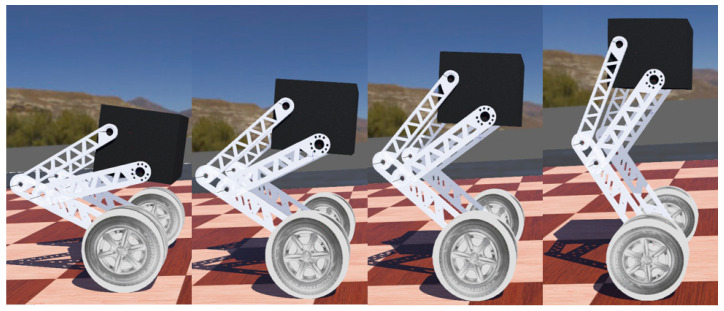
Highly varying state.

In order to validate the control performance of the currently designed control system for the four-link wheel-legged robot, a model file was integrated into Webots. Additionally, two slopes were configured in the simulation environment to demonstrate the robot’s current motion capabilities. The robot is capable of adjusting its height between a lower and higher position, with a maximum height variation of 15 cm [34], see Figure 12.

**Figure 13:**
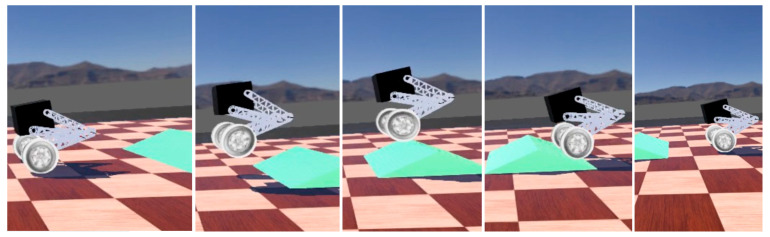
The robot accelerates through the small slope state.

The small slope and a long slope of 20° are set in the environment, and the machine is allowed to move forward and actively adjust the attitude. As shown in Figure 13, the robot passes the small slope at a high speed and can land stably.

**Figure 14:**
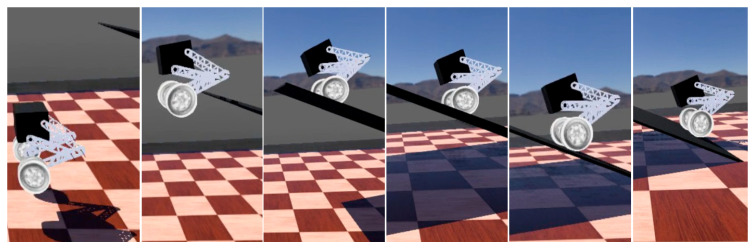
The robot is in an uphill state.

As shown in Figure 14, in the uphill phase, the robot actively changes the inclination angle of the body, puts the center of gravity forward, and reduces the speed uphill. At the same time, when falling from the slope, the robot can also adjust the angle to buffer the landing.

It can be seen that the stability of the robot under the LQR algorithm is guaranteed, and the stability of the body can be maintained under the complex terrain set by the simulation environment.

## 6. Experiments and Analysis of Results

The LQR-PD control algorithm is tested on an experimental platform, comparing traditional control methods and analyzing position, velocity, and attitude control performance.

### 6.1. Experimental Platform for Balance Control of Bipedal Wheel-Legged Robot

**Figure 15:**
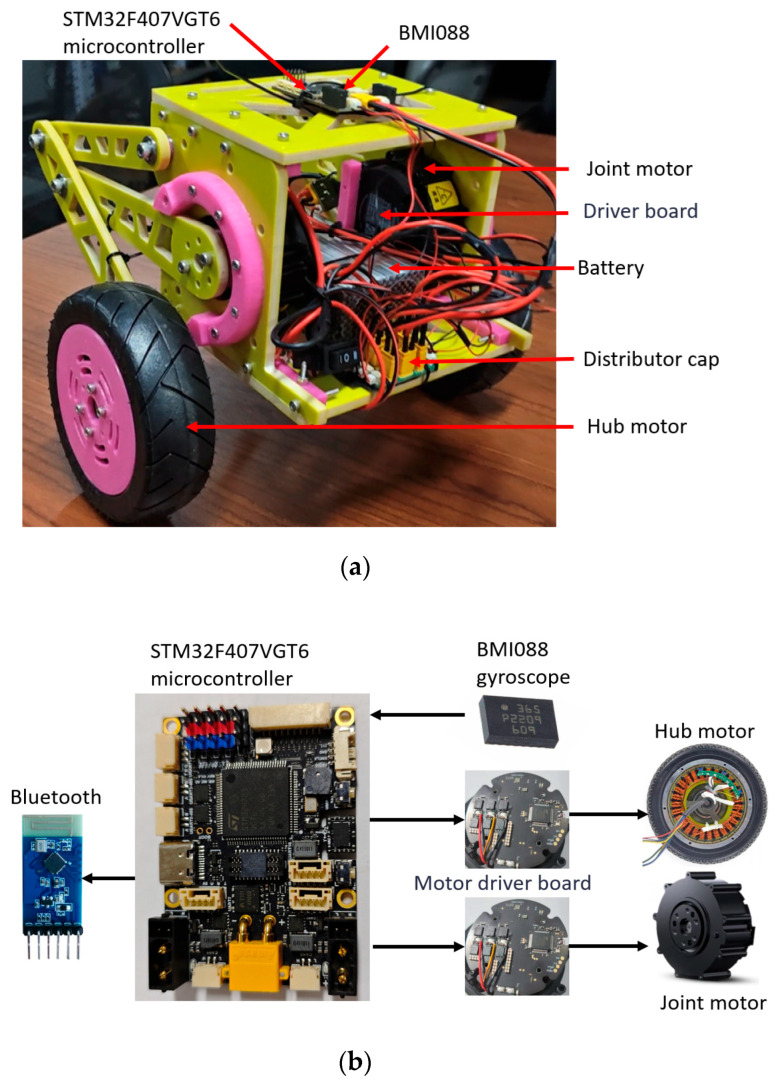
Photographs of the robot. (**a**) Physical robot; (**b**) Hardware connection diagram.

To verify the proposed LQR-PD hybrid control method, we designed a bipedal wheel-legged robot and built a hardware and software platform. The robot can maintain a horizontal body posture while its height varies from 0.3 m to 0.45 m, and its mass is approximately 7 kg. The platform consists of the robot’s main body, an inertial measurement unit sensor module, a remote-control Bluetooth module, and a real-time control module, with a data acquisition and control frequency of 1000 times per second. Our self-designed robot can achieve the movement of wheels and the adjustment of the posture of the leg structure composed of four links. The inertial measurement unit uses the BMI088 six-axis sensor to obtain positioning data at a reading frequency of 2000 Hz and a resolution of 0.004°/s. The Bluetooth module adopts JDY-33, which can achieve data transmission in the 2.4 GHz working frequency band, with a maximum RF power output of 6 dBm and an effective communication radius of 30 m. For details of the platform components, see Figure 15. The balance control of the entire machine is mainly determined by the in-wheel motor, and the height change in the entire machine is determined by the joint motor. The motor parameter information is shown in Table 3.

The main controller receives the data detected by the IMU, parses it using the LQR-PD algorithm, and transmits it via the CAN protocol to the motor as the control input for the motor. The motor encoder then transmits the actual motor speed and position data back through CAN to the main controller for feedback control calculation, forming a closed-loop control program.

### 6.2. Robot Control Experiment

To verify the balance performance of the robot, an external thrust disturbance was manually applied to the robot in a stable equilibrium state, and the recovery ability of its pitch angle was observed.

**Figure 16:**
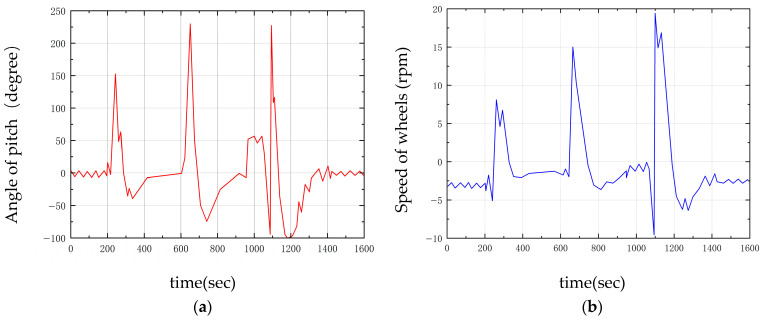
The parameter change in the disturbance state. (**a**) Angle of pitch; (**b**) Speed of wheels.

The experimental results show that the pitch angle of the robot increases sharply at the moment of disturbance, as shown in Figure 16a, and the robot moves backward. Subsequently, the robot quickly adjusts the wheel speed, as shown in Figure 16b, to achieve a rapid recovery of the pitch angle. Multiple disturbance tests indicate that the system has excellent dynamic response capabilities.

In the movement steering control experiment, tests were conducted under the established control strategy, focusing on the dynamic characteristics of the robot during turning.

**Figure 17:**
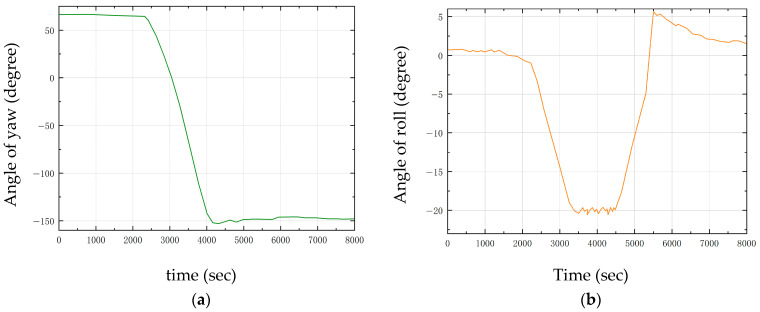
Variation of bending parameters. (**a**) Angle of yaw; (**b**) Angle of roll.

When the robot initiates a left turn at the 2nd second, the yaw angle rapidly changes from 50° to −150° and stabilizes after the turn is completed at the 4th second, as shown in Figure 17a. Meanwhile, to counteract the centrifugal force generated during turning, the contraction of the robot’s left leg causes the roll angle to deviate from the initial 0° to −20° and gradually recover after the turn is finished, as shown in Figure 17b. The experimental results demonstrate that the control strategy can effectively ensure the attitude balance of the robot during dynamic movement.

### 6.3. Experiments and Result Analysis of LQR-PD Control

To validate the effectiveness of the LQR-PD control algorithm in bipedal wheel-legged robot control, comparative experiments were conducted against the PID-PD control algorithm, with the latter serving as the baseline. The experiments involved applying external disturbances to test the robot’s ability to recover balance while analyzing variations in state variables, including displacement, velocity, and joint angles.

**Figure 18:**
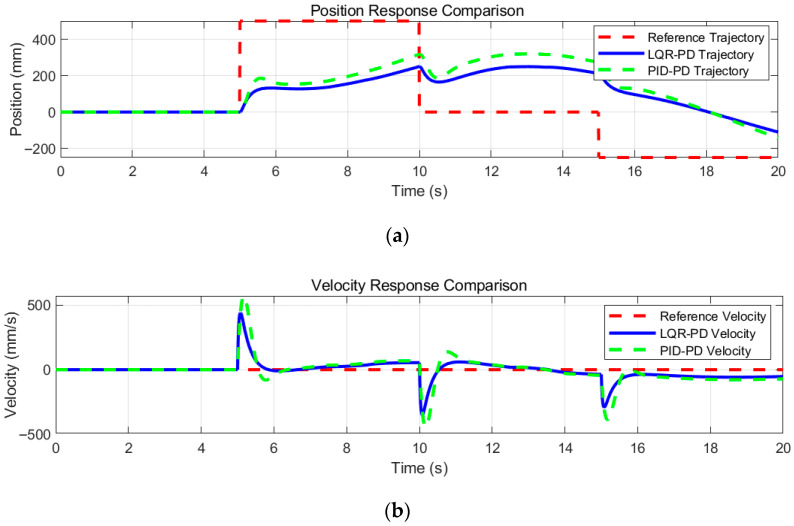

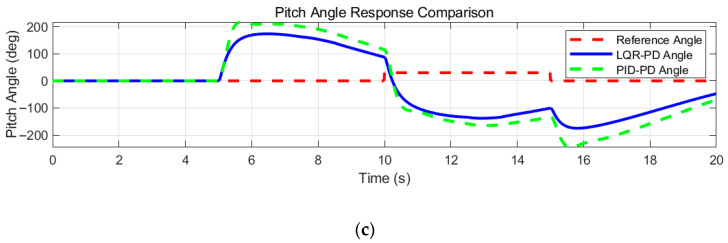
Experimental results of horizontal stability anti-jamming: (**a**) Position; (**b**) Velocity; (**c**) Pitch angle.

As shown in the figure, disturbances were applied to the robot during its equilibrium state at 5 s, 10 s, and 15 s to observe the convergence speed and amplitude of the two control algorithms, see Figure 18 and Table 4.

Experiments show that LQR-PD has remarkable advantages in controlling bipedal wheel-legged robots. In position tracking, its RMSE is about 1% lower and ITAE over 30% smaller than those of PID-PD, with less dynamic error accumulation. For velocity control, RMSE and ITAE are only 69.1% and 60.5% of PID-PD’s, respectivley, featuring smaller fluctuations and a faster response. In pitch angle control, RMSE and ITAE decrease by over 20% and 36.8%, respectivley, ensuring better attitude stability. With RMSE and ITAE introduced, position error drops by 1–20.5%, and dynamic response efficiency rises by 30.6–36.8%, providing a standard for algorithm comparison, verifying complex terrain adaptability. It outperforms PID-PD in tracking precision, stability, and robustness comprehensively.

## 7. Conclusions

This study investigates motion control methodologies for wheel-legged robots through a comprehensive review of the domestic and international literature. Focusing on the nonlinear, strongly coupled, and underactuated characteristics of a four-bar linkage wheel-legged robot, we propose a hybrid control system integrating LQR with PD and VMC for wheel leg height compensation. This approach addresses challenges including complex mathematical modeling, high computational demands, and degraded control performance due to unmodeled centroid height variations. Simulation analyses conducted in Simulink and Webots demonstrate that the four-bar linkage wheel-legged robot achieves climbing capability on 20° slopes, obstacle clearance up to 15 cm in height, and longitudinal jumping distances of 1.2 m, significantly enhancing its obstacle negotiation capacity and overall performance. Experimental tests on disturbance rejection reveal that the LQR-PD control algorithm exhibits superior balance recovery capability compared to that of PID-PD. The four-bar linkage design broadens potential application scenarios for wheel-legged robots.

Future works in this study area may focus on the following: (1) Exploring hardware upgrades to achieve temperature and pressure resistance; (2) Designing a hierarchical control strategy for SMC, combining it with the existing LQR-PD framework, and leveraging the robustness of SMC to achieve high-level anti-interference capabilities; (3) Realizing dynamic obstacle avoidance in extreme conditions through visual SLAM technology; and (4) Enhancing the adaptive control capability against external disturbances in extreme environments and improving the robot’s motion stability under high-temperature, dusty, or complex geological conditions.

## Figures and Tables

**Table 1:** Parameter definitions of inverted pendulum model.

Symbol	Meaning, Unit
M	Body mass (kg)
m	Wheel mass (kg)
l	Distance from body center of mass to wheel center (m)
r	Wheel radius (m)
D	Distance between left and right wheels (m)
g	Gravitational acceleration (m/s^2^)
$\theta ,\dot{\theta ,}\ddot{\theta }$	Machine pitch angle (rad), angular velocity (rad/s), angular acceleration (rad/s^2^)
$T_{L},T_{R}$	The driving torque of the wheel (N·m)
$N_{L},N_{R},P_{L},P_{R}$	The force between the body and the wheel in the x and y directions (N)
$F_{L},F_{R}$	The friction between the wheel and the ground (N)
$x_{L},x_{R},{\dot{x}}_{L},{\dot{x}}_{R},{\ddot{x}}_{L},{\ddot{x}}_{R}$	Wheel displacement m, velocity (m/s), acceleration (m/s^2^)
$\omega_{L},\omega ,{\dot{\omega }}_{L},{\dot{\omega }}_{R}$	Wheel rotation angular velocity (rad/s), acceleration (rad/s^2^)
I	Wheel moment of inertia (kg·m^2^)
$J_{x},{J_{y},J}_{z}$	Moment of inertia of the body around the *x*,*y*,*z*-axis (kg·m^2^)
$\delta ,\dot{\delta },\ddot{\delta }$	Course angle (rad), angular velocity (rad/s), angular acceleration (rad/s^2^)

**Table 2:** Parameter definitions of the wheel-legged robot model.

Symbol	Meaning, Unit
$l_{1},l_{2},l_{3},l_{23}$	The length of the corresponding rod (m)
$l_{4}$	The distance between the joints (m)
$l_{5}$	Distance (m) from the intersection point of the upper joint to the lower lever (m)
$l_{leg}$	Pendulum height (m)
$\theta_{1}$	The angle between bar $l_{2}$ and the horizontal axis (rad)
$\theta_{2}$	Angle between auxiliary connecting rod $l_{4}$ and $l_{5}$ (rad)
$\theta_{3}$	Angle between auxiliary connecting rod $l_{5}$ and $l_{23}$ (rad)
$\theta_{4}$	The angle between bar $l_{1}$ and the horizontal axis (rad)
$\theta_{5}$	The angle between bar $l_{23}$ and the horizontal axis (rad)
$\theta_{6}$	The angle between bar $l_{3}$ and the horizontal axis (rad)
$L_{desired}$	Desired leg length (m)
$x_{c2},x_{c1},x_{c3},x_{c23}$	Vertical coordinates of the center of mass of the connecting rod
$L_{0}$	Height of the total center of gravity of the system (m)
$l_{w}$	Height of the center of gravity of the pendulum (m)
R	Drive wheel radius (m)
$m_{w}$	Drive wheel mass (kg)
$m_{b}$	Body mass (kg)
$m_{l}$	Pendulum mass (kg)
$m_{p}$	Total system mass (kg)
$L_{1}$	Distance from center of gravity to wheel axle (m)
$I_{w1}$	Drive wheel moment of inertia (kg·m^2^)
$I_{p1}$	Moment of inertia of a pendulum (kg·m^2^)
N	Normal force (N)
P	Combined force of gravity and acceleration (N)
T	Driving torque (N·m)

**Table 3:** Parameters of the motors.

Motors	Rated Torque (N·m)	Rated Speed (RPM)	Power (W)	Control Frequency (Hz)
Joint Motors	12	196	150	1000
In-wheel Motors	2	120	103	1000

**Table 4:** Experimental analysis of horizontal stability anti-jamming.

Type	Method	Mean	Variance	RMSE	ITAE
Position (mm)	LQR-PD PID-PID	38.3085 66.5343	62,364.4706 63,661.0724	249.73 252.31	1285.6 1852.4
Velocity (mm/s)	LQR-PD PID-PID	−5.2762 −6.9331	4958.3190 10,378.1216	70.42 101.87	542.1 896.3
Pitch angle (10*deg)	LQR-PD PID-PID	−30.1684 −37.6911	13,543.1260 21,434.8167	116.37 146.41	987.5 1562.7

## Data Availability

All date are contained within the article.

## Abbreviations

Abbreviations	Full name
WBRs	Wheeled Bipedal Robots
LQR	Linear Quadratic Regulator
VMC	Virtual Model Control
PID	Proportional-Integral-Derivative
PD	Proportional-Derivative
CG Height	Center of Gravity Height
IDA-PBC	Interconnection and Damping Distribution-Passive Control
UKF	Unscented Kalman Filter
IMU	Inertial Measurement Unit
CAN	Controller Area Network
RMSE	Root Mean Square Error
ITAE	Integral of Time-Weighted Absolute Error
